# Viability Assessment Following Anticancer Treatment Requires Single-Cell Visualization

**DOI:** 10.3390/cancers10080255

**Published:** 2018-08-01

**Authors:** Razmik Mirzayans, Bonnie Andrais, David Murray

**Affiliations:** Department of Oncology, Cross Cancer Institute, University of Alberta, Edmonton, AB T6G 1Z2, Canada; bonnie.andrais@ahs.ca (B.A.); david.murray5@ahs.ca (D.M.)

**Keywords:** single-cell analysis, high throughput assays, solid tumor-derived cell lines, cisplatin, ionizing radiation, MTT

## Abstract

A subset of cells within solid tumors become highly enlarged and enter a state of dormancy (sustained proliferation arrest) in response to anticancer treatment. Although dormant cancer cells might be scored as “dead” in conventional preclinical assays, they remain viable, secrete growth-promoting factors, and can give rise to progeny with stem cell-like properties. Furthermore, cancer cells exhibiting features of apoptosis (e.g., caspase-3 activation) following genotoxic stress can undergo a reversal process called anastasis and survive. Consistent with these observations, single-cell analysis of adherent cultures (solid tumor-derived cell lines with differing p53 status) has demonstrated that virtually all cells—irrespective of their size and morphology—that remain adherent to the culture dish for a long time (weeks) after treatment with anticancer agents exhibit the ability to metabolize 3-(4,5-dimethylthiazol-2-yl)-2,5-diphenyl- tetrazolium bromide (MTT). The purpose of this commentary is to briefly review these findings and discuss the significance of single-cell (versus population averaged) observation methods for assessment of cancer cell viability and metabolic activity.

## 1. Introduction

Cell-based assays are indispensable for preclinical evaluation of agents with potential anticancer properties. In a typical anticancer drug discovery approach, the test compound is first evaluated in a short-term, high throughput colorimetric/fluorimetric assay in a multiwell plate format to determine the IC_50_ value (drug concentration resulting in 50% inhibition of the selected screening parameter). The IC_50_ is often interpreted to reflect 50% “killing”, perhaps because multiwell plate kits are marketed as assays for viability/cytotoxicity. Traditionally, the compound is further tested in a battery of assays for apoptotic signaling, such as caspase activation, cytoplasmic phosphatidylserine (PS) externalization, and mitochondrial membrane potential (MMP) disruption. Finally, the compound is tested in a more laborious and time-consuming colony formation assay which has emerged as a gold standard for assessment of cellular radiosensitivity and chemosensitivity. This conventional battery of cell-based tests, or only the gold standard colony formation assay, is also widely used to identify pairs of compounds (e.g., small molecule inhibitors) for synthetic lethal interactions. Synthetic lethality occurs when perturbing two or more cellular targets simultaneously results in loss of viability, whereas perturbing only one of these targets does not [1].

**Figure 1:**
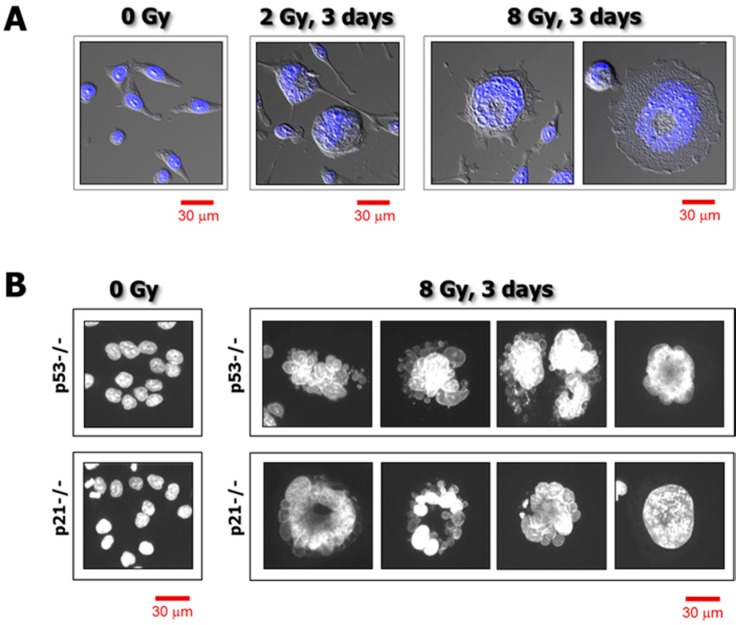
(**A**) Confocal microscopy images showing nuclear morphology of p21 knockout HCT116 cells before and 3 days after exposure to γ radiation. Nuclear content (DAPI staining) is shown in blue. (**B**) Fluorescence images showing the nuclear morphology of p53 knockout (p53−/−) and p21 knockout (p21−/−) HCT116 cells before and 3 days after irradiation. Nuclear content (DAPI) is shown in white. Data obtained from Mirzayans et al. [3].

A shortcoming of such an approach is the reliance on cell population-based analysis. All of the aforementioned short-term assays depend on using large numbers of cells and averaging the measured response parameters. Despite their relative ease of performance and high throughput (e.g., using a plate reader to generate “viability” data for a large number of samples simultaneously), all global analyses have the disadvantage of obscuring the presence of functionally important subpopulations. In the context of cancer therapy, we recently discussed the potential significance of such subpopulations [2]. Notably, the development of polyploid/multinucleated giant (hypertrophic) cells (Figure 1) that are often scored as “dead” in such assays is a major confounding factor. Giant cells developed in response to genotoxic stress do not form a macroscopic colony (aggregates of >50 cells) in the time span of the conventional clonogenic assay (~10 days). However, most giant cells generated in response to moderate, clinically relevant doses of anticancer agents remain viable and secrete tumor promoting factors, while also having the potential of giving rise to highly metastatic and therapy-resistant progeny.

The intent of this commentary is to underscore the value of single-cell analysis for radiosensitivity/chemosensitivity assessment with solid tumor-derived cell lines. Specifically, we briefly describe the assays optimized by us [3,4,5] and highlight key observations made with cancer cells treated with ionizing radiation and chemotherapeutic drugs. We also briefly review compelling experimental data reported in the past decade that highlight the importance of single-cell observation methods for: (i) distinguishing between dying cells (e.g., cells with activated caspase cascade) and dead cells; and (ii) demonstrating the ability of cancer cells to recover from the brink of apoptotic cell death. 

## 2. Comparing Proliferation Arrest and Colony Formation Assays for Genotoxicity Assessment

As mentioned above, cell population-based assays are widely used for initial assessment of new compounds with potential anticancer properties and for comparing the degree of sensitivity of different cell cultures to a given genotoxic agent. In such assays, determining a 50% effect—so-called IC_50_ for chemical compounds and ID_50_ (inhibiting dose, 50%) for physical agents—is considered an informative indicator of cytotoxicity/antiproliferative activity of clinical relevance. Of several cell-based assays used by us over the years (since the early 1980’s [6]), determining the degree of proliferation arrest post-treatment by direct cell counting proved to be most informative and versatile [3,4,5]. 

**Figure 2:**
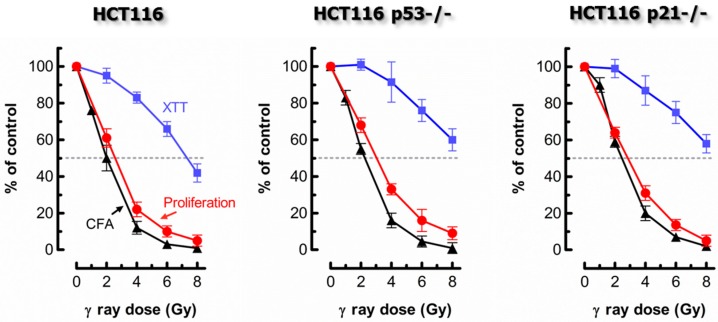
Comparing the responses of the HCT116 parental cell line and its p53 knockout (HCT116p53−/−) and p21 knockout (HCT116p21−/−) derivatives to ionizing radiation. Radiosensitivity was measured by the proliferation (cell counting) (red circles), colony formation (black triangles), and 96-well plate XTT colorimetric (blue squares) assays. The results were generated by exposing cells to radiation followed by incubation in fresh medium for 3 days (proliferation; XTT) or 10 days (colony formation). Bars, standard error (SE); CFA, colony forming ability. XTT, 2,3-bis-(2-methoxy-4-nitro-5-sulfophenyl)-2H-tetrazolium-5-carboxanilid). Data obtained from Mirzayans et al. [3,4].

The assay optimized by us is typically completed in 4 days from the seeding of cells [3,4,5]. Despite being short-term, it generates radiosensitivity/chemosensitivity results that are similar to those obtained by the longer-term and considerably more labor-intensive colony formation assay. To date, we have compared these two assays for radiosensitivity assessment in twelve solid tumor-derived cell lines [3,4] (also see Figure 2), and for sensitivity to 254-nm ultraviolet light (UV) and the chemotherapeutic drug oxaliplatin in the HCT116 colon carcinoma cell line and its p53 knockout derivative (data to be published elsewhere). In all cases, for a given cell line, the ID_50_ (ionizing radiation; UV) or IC_50_ (oxaliplatin) values obtained by the two assays were virtually identical.

## 3. Single-Cell Analysis of Cancer Cell Response to Genotoxic Stress

In addition to reproducibility and relative ease of performance, our optimized assay for inhibition of proliferation by cell counting enables concurrent single-cell evaluations to assess different parameters in the same cultures under identical experimental conditions. In most experiments, for a given cell line, we prepare several sets of dishes to determine the impact of a DNA-damaging agent not only on cell proliferation, but also on cell morphology, nuclear content, viability, and metabolic ability.

**Figure 3:**
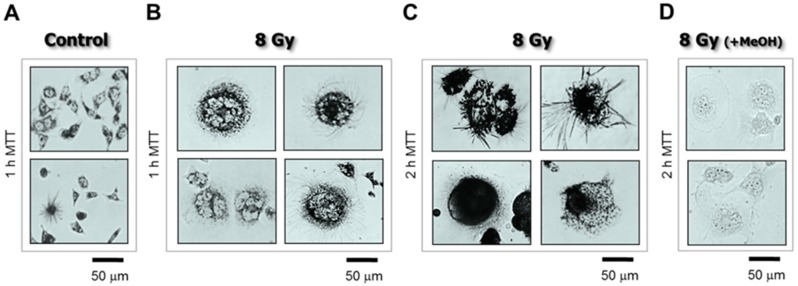
Bright-field microscopy images depicting the metabolic activity of HCT116p53−/− cultures before (**A**) and 3 days after 8-Gy irradiation (**B**–**D**). Metabolic activity was measured by the ability of the cells to convert the yellow 3-(4,5-dimethylthiazol-2-yl)-2,5-diphenyl-tetrazolium bromide (MTT) to its purple formazan metabolite, appearing as dark granules and crystals. Cells were incubated with MTT (0.5 mg/mL) for 1 h (**A**,**B**) or 2 h (**C**,**D**). As a negative control, cells in some dishes were first treated with methanol to inhibit their metabolic activity, and then incubated with MTT (**D**). Data obtained from Mirzayans et al. [3].

Cell morphology can be assessed by various staining protocols, including immunostaining. To this end, we use a β-actin-specific antibody to visualize cell morphology, and DAPI (4′,6-diamidino-2-phenylindole) counter-staining of DNA to assess nuclear morphology/content [2,3]. For cell viability, we perform the trypan blue-exclusion assessment of adherent cells without exposing them to trypsin [3,5]; exposure to trypsin to detach the cells followed by preparation of cell suspensions and mixing with the trypan blue solution can result in false positive staining of some cells due to transient cell membrane injury. For metabolic activity, we perform microscopic evaluation of the ability of individual cells to convert the tetrazolium salt 3-(4,5-dimethylthiazol-2-yl)-2,5-diphenyl-tetrazolium bromide (MTT) to its water-insoluble formazan derivative [3,4,5]. MTT formazan metabolites are visualized as purple intracellular granules and crystals under a light microscope (Figure 3).

Among these parameters, cell counting (proliferation) and trypan blue-exclusion assessments need to be undertaken soon after completion of the experiment (e.g., 3 days post-irradiation). For immunostaining and the MTT assay, however, on the experiment-completion day, cells are fixed and stored in methanol (for immunostaining), or incubated with MTT (for 1–2 h), air dried, and mounted with glycerol; these samples can then be processed/evaluated when convenient (days or weeks later). Such an approach enables one operator to compare the responses of several cell lines to a given anticancer agent in a single scoring session.

Employing this strategy, we recently reported the responses of a number of widely-used solid tumor-derived cell lines to moderate doses of ionizing radiation (between 2 and 8 Gy) typically used in the colony formation assay [3,4]. The study involved cell lines that express wild-type p53 (e.g., MCF7 breast carcinoma; HCT116 colon carcinoma), or mutant p53 (e.g., MDA-MB-231 breast carcinoma), or those which do not express p53 (e.g., HCT116p53−/− colon carcinoma; SKOV3 ovarian carcinoma). We demonstrated that: (i) γ ray exposure followed by incubation for 3 days resulted in sustained proliferation arrest, which was attributed in part to the development of highly enlarged cells that remained adherent to the culture dish; (ii) enlarged cancer cells had a massive cytoplasmic content, the majority of which contained either a highly enlarged nucleus (reflecting polyploidy) or multiple nuclei; and (iii) virtually all cells that remained adherent to the culture dish for long times (up to 3 weeks) post-irradiation maintained cell membrane integrity, as determined by trypan blue exclusion, and exhibited the ability to metabolize MTT. 

Employing this approach, we have also determined the responses of representative cancer cell lines to moderate, clinically relevant doses of cisplatin [5] and oxaliplatin (unpublished observations). The results were similar to those obtained following γ ray exposure. In MDA-MB-231 breast carcinoma cells, for example, after treatment with doses of each of these drugs (e.g., 10 µM, 3 days) that resulted in sustained proliferation arrest in ~90% of cells (i.e., ~IC_90_), virtually all cells remained adherent to the culture dish and rapidly converted MTT to its water-insoluble metabolite ([5], and unpublished observations). The majority of proliferation-arrested cells exhibited a highly enlarged morphology ([5], and unpublished observations).

It is noteworthy that the conventional multiwell plate colorimetric (XTT) and/or fluorimetric (CellTiter-Blue) assays were also included in some experiments. The degree of radiosensitivity [3,4] and chemosensitivity [5] as measured by these cell population-based assays was markedly skewed towards resistance when compared to the responses measured by the cell counting (proliferation) assay (also see Figure 1). Such a discrepancy is not surprising given that the single-cell MTT, coupled with image analysis protocol, demonstrated that the level of metabolic activity per cell can be strikingly greater (e.g., ~10 times in some cell lines) for γ radiation-exposed or drug-treated cultures, which contained a high proportion of enlarged cells, than for sham-treated controls [4,5].

**Figure 4:**
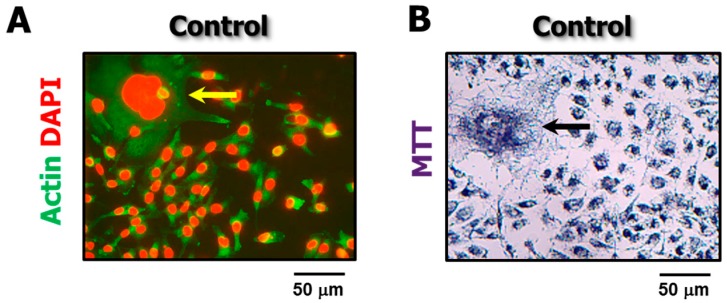
Representative fluorescence (**A**) and bright-field microscopy (**B**) images showing the presence of “enormous” cells (arrows) in proliferating cultures of the MDA-MB-231 cell line in the absence of exogenous stress.

The proportion of enlarged cells in a culture of a given cell line depends on several factors, including the genetic background of the cells, as well as the type and amount of genotoxic stress. For moderate doses of anticancer agents used by us (e.g., doses resulting in ~90% proliferation arrest), we found that exposure to ionizing radiation followed by incubation for 3 days resulted in enlargement of the majority (>50%) of cells within cultures of all 12 cell lines that we have examined [3,4]; some enlarged cells became “fatter” with increasing post-irradiation incubation time [3]. In response to chemotherapeutic drugs, however, we found that some cell lines (e.g., MDA-MB-231 and MCF7 breast carcinoma) exhibited a much higher proportion of enlarged cells than others (e.g., HCT116). Furthermore, cultures of some cell lines (e.g., MDA-MB-231) contain a subset of “enormous” cells even without exposure to exogenous stress [2] (Figure 4).

In short, highly enlarged cancer cells (reflecting premature senescence/polyploidy/multinucleation) contribute to sustained proliferation arrest following treatment with DNA-damaging agents, but not all proliferation-arrested cells are enlarged. Cells activating transient cell cycle checkpoints (a well-studied pro-survival response) and those triggered to undergo apoptosis and/or autophagy also contribute to proliferation arrest but are unlikely to exhibit an enlarged morphology, compared to the bulk of pretreated cells. 

## 4. Surviving Stress-Induced Apoptotic Signaling 

Induction of apoptosis [7] or autophagy [8] in cancer cells following genotoxic stress is not always associated with their demise. To this end, advances in single-cell assays have focused attention on the fact that intracellular biochemical fluctuations can have profound effects on phenotype (survival versus death) (reviewed in [7,9,10]). These fluctuations cause genetically identical cells (e.g., different subsets of cells within a culture of a given human cell line) to differ significantly in their responsiveness to cytotoxic stimuli even in a uniform environment [11,12,13,14,15,16,17,18,19,20]. Below we will briefly discuss the multiple (and opposing) properties of caspase-3 and reversibility of the apoptotic cascade. In view of these rather unexpected discoveries, we will also point out the practical aspects of the single-cell MTT assay for cancer cell viability assessment.

### 4.1. Apoptotic and Non-Apoptotic Functions of Caspase-3

Apoptosis derives from the Greek for “dropping off”, and refers to a highly complex and sophisticated programmed cell death process involving an energy-dependent cascade of molecular events. Apoptosis can be initiated by a plethora of stimuli that generally feed into one of two distinct but overlapping signaling pathways—the extrinsic or death receptor pathway, and the intrinsic or mitochondrial pathway [21]. In addition, a perforin/granzyme-dependent apoptotic pathway has been identified, which is used by effector lymphocytes to eliminate transformed or virus-infected cells [21]. The extrinsic, intrinsic, and granzyme B pathways converge on the same terminal or execution phase. This execution phase is mediated by active caspase-3. As such, caspase-3 activation has been widely used as a surrogate marker for apoptosis (e.g., [22,23,24]). 

Given its pivotal role in the execution phase of apoptosis, caspase-3 is expected to function as a tumor suppressor. Several recent studies, however, have shown that instead of functioning as a tumor suppressor, active caspase-3 plays an important role in carcinogenesis, metastasis, and therapy resistance, at least in solid tumors. Zhou et al. [25], for example, reported recently that active caspase-3 promotes metastasis and underlies therapy resistance in the HCT116 colon carcinoma model. Specifically, in vitro, caspase-3 knockout HCT116 cells were significantly less clonogenic in the soft agar assay, less invasive, and more sensitive to anticancer agents than parental (caspase-3-expressing) cells. In vivo, caspase-3 knockout cells were significantly more sensitive to radiotherapy and were also less prone to pulmonary metastasis when inoculated either subcutaneously or intravenously in nude mice. Consistent with these properties, caspase-3 was shown to promote epithelial to mesenchymal transition (EMT) [25], which is now accepted as a central mechanism that induces invasion and metastasis of tumors [26,27]. Therefore, Zhou et al. [25] proposed that “therapeutic targeting of caspase-3 may not only increase the sensitivity of cancer cells to chemotherapy and radiotherapy, but also inhibit cancer cell invasion and metastasis.” Several previous reports with different tumor models [28,29,30,31] and with cancer patients [24,32] have led to a similar conclusion. Huang et al. [28], for example, showed that caspase-3 can promote tumor repopulation after radiotherapy through a paracrine signaling pathway (called “Phoenix Rising”) involving prostaglandin E_2_ (also see [33,34,35]).

### 4.2. Reversibility of Apoptosis in Solid Tumor-Derived Cell Lines

In the past decade it has also become evident that cancer cells can recover from the brink of apoptotic cell death through a process called anastasis (“rising to life” in Greek). This observation was first reported by Tang et al. in 2009 [36], and subsequently confirmed by the same group [37,38,39,40] and independently by Sun et al. [41]. Employing time lapse microscopy coupled with a battery of single cell assays, it was shown that treatment of cancer cell lines with an apoptosis-triggering agent resulted in caspase-3 activation, PS externalization (visualized by Annexin V staining), cell shrinkage, and other features of apoptosis (i.e., plasma membrane blebbing, cytoplasmic condensation, apoptotic body formation, mitochondrial fragmentation, mitochondrial cytochrome *c* release, DNA/chromatin condensation, and nuclear fragmentation). Upon removal of the apoptosis-inducing agent and incubation in fresh culture medium, the cells with apoptotic features gradually recovered, regained “normal” (pretreatment) morphology, and resumed proliferation. Anastasis has been observed following treatment with different cytotoxic agents, including ethanol, dimethyl sulfoxide, staurosporine, and taxol [39]. Recently, Sun et al. [41] have demonstrated that anastasis is a two-stage program. During the early stage, cancer cells transition from a proliferation-arrested state to a proliferating one, and in the late stage they switch from proliferation to migration. The molecular signatures of these processes are beginning to emerge [40,41]. It is important to note that all studies on anastasis to date have been performed with adherent cells.

### 4.3. The Single-Cell MTT Viability Assay: Simple and Sensitive

As discussed previously [33], numerous reports have drawn conclusions on apoptotic cell death based only on averaged molecular readouts in a population of cells, such as measurement of active caspase-3 and upregulation of proapoptotic factors (e.g., PUMA, NOXA and BAX), without actually determining cell demise. Of particular note, caspase activation, determined in a multiwell plate format, has led to the assumption that multiwell plate colorimetric/fluorimetric “viability” assays measure loss of viability (i.e., cytotoxicity) after genotoxic treatment (e.g., [42,43]). The aforementioned recent discoveries, however, underscore the importance of distinguishing between dying cells (exhibiting activated apoptotic cascade) and dead cells. 

Our studies have demonstrated that, unlike the widely-used multiwell plate assays, the single-cell MTT viability assay is a powerful tool for distinguishing between dying and dead cells [2,3,4,5]. The MTT reagent is a cell membrane impermeable tetrazole [44]. The reagent is taken up by cells through endocytosis [45] and is reduced to its purple insoluble formazan metabolites by NAD(P)H-dependent oxidoreductase enzymes, largely in the cytosolic compartments [44,46]. Unlike other tetrazolium salts that are widely used in colorimetric assays, the MTT reagent does not require an electron coupling step to facilitate its uptake by the cells; the net positive charge on MTT is sufficient for its uptake via the plasma membrane potential [44]. By virtue of these properties, MTT is particularly useful for assessment of viability of individual cells by microscopy in a simple and yet highly informative and reproducible manner. 

The single-cell MTT assay is simple because it merely involves adding the MTT reagent to the culture medium, incubating the cells for 1–2 h, and then performing microscopic evaluation and acquiring cell images. The data presented in Figure 3 were generated following the protocol outlined below (reproduced from [3]):

- Cells were plated in 35 mm dishes (~20,000 cells/2 mL medium/dish) and incubated overnight.
- The cells were then either exposed to γ radiation or sham-irradiated (controls) and incubated for 3 days.
- The medium was replaced with fresh medium containing MTT (final concentration, 0.5 mg/mL) and the cells were returned to the incubator.
- After incubation with MTT for 1–2 h, the cells were viewed under a microscope (bright-field; 20× objective) and their images acquired.

Data evaluation is also straightforward. Irrespective of the level of the metabolic activity of a cell, if the cell is capable of converting MTT to its formazan metabolite to give rise to dark granules/crystals, then it cannot be dead. Therefore, assuming that a particular genotoxic treatment might result in a decrease in the metabolic activity of a subset of cells (e.g., reflecting changes in the oxidoreductase enzymes), these cells will still be capable of metabolizing MTT, albeit at a slower rate (e.g., within ~2 h) compared to control (sham-treated) cells. On the other hand, dead cells will not metabolize MTT and thus remain clear of tetrazolium granules and crystals [3] (also see Figure 3D).

Images obtained in the single-cell MTT assay can be used to estimate the degree of metabolic activity of cells irrespective of their size (for details, see [4,5]). To this end, bright field microscopy images, such as those shown in Figure 3, are converted to gray scale and then inverted; this results in dark (black) backgrounds and bright (white) signals (reflecting cellular MTT formazan granules and crystals) that are ideal for image analysis. 

Since our published studies [3,4,5], we have determined that MTT-treated samples can be stored in glycerol. To this end, after completion of cell incubation with MTT, the medium is removed and cells are air dried. A small drop (~20 µL) of glycerol/PBS (9:1 vol/vol) mounting medium is placed in the middle of each dish, and a coverslip is carefully lowered onto the drop of mounting medium in such a way as to prevent the formation of bubbles. Storing cells under these conditions for several weeks did not noticeably alter the intensity of intracellular MTT formazan granules and crystals.

The single-cell MTT protocol as described above circumvents several shortcomings of other viability assays that rely on cellular uptake of large dyes, which are also performed at the single-cell level. We will only consider trypan blue, which has emerged as the most popular large dye for viability assessment. The first (and perhaps most important) shortcoming is the creation of false positives, as pointed out by Husmann in a Letter to the Editor of Cell Death and Differentiation [47]. Cells with an intact plasma membrane exclude trypan blue (and other large dyes), whereas cells with compromised plasma membranes rapidly take up trypan blue and are visualized as dark blue under light microscopy. However, in 2013, Husmann [47] reviewed some key articles demonstrating that injuries of the plasma membrane can be transient, which may result in an uptake of large dyes, and hence create false positives. The second shortcoming is that the cells must be evaluated in a very short time frame (minutes) after incubating them with trypan blue, which limits the number of samples that can be tested in a single scoring session. The third shortcoming is that trypan blue-treated samples cannot be stored for future evaluation. 

## 5. Conclusions

The value of single-cell analysis has long been appreciated for different biological systems, ranging from microorganisms [48] to human cells [49,50,51,52]. There have been excellent reviews on the clinical implications of tumor heterogeneity and methodologies for its study at the single cell level (e.g., [50]). In the field of cellular responses to genotoxic stress, however, most investigators rely on assays that determine averaged responses in a population of cells. In particular, the use of multiwell plate colorimetric/fluorimetric assays appears to have become the “standard” for cancer cell viability/cytotoxicity assessment following treatment with genotoxic agents. The potential for misinterpretation of results obtained by cell population-based assays has been discussed by us [5] and others [53]. 

Employing single cell analysis, we have reported that anticancer agents at nontoxic levels trigger the development of highly enlarged cells—reflecting premature senescence/polyploidy/multinucleation—in solid tumor-derived cell lines with differing p53 status [3,4,5]. This observation is not surprising given the wealth of experimental data on stress-induced cancer cell enlargement reported from different laboratories since the 1950s (reviewed in [2,34]). However, our observation that a significant proportion of enlarged cells remain viable (e.g., exclude large dyes; metabolize MTT) for long periods of time (weeks) post-treatment is potentially alarming, given that a single giant cancer cell has been reported to be sufficient to cause metastatic tumors in a mouse model [54].

The complex life cycle experienced by “giant” cancer cells, ultimately resulting in the emergence of therapy-resistant progeny with stem cell-like properties, has led to the atavistic model of cancer [55]. As pointed out by Thomas et al. [56], in the atavistic model, “cancer is interpreted as a reversion to phylogenetically prior capabilities, namely the release of a highly conserved survival program encrypted in every eukaryotic cell, and hence in every multicellular animal. This ancient program would have evolved during the pre-Cambrian period, when the selective forces acting on unicellular organisms were favoring adaptations prioritizing the continuation/proliferation of cellular life in different, often adverse, environmental conditions.” Mounting evidence supports the stress-induced atavistic reprogramming (STAR) [56] model for both cancer development and the acquisition of therapy resistance (e.g., [55,56,57,58,59]). 

In the current article, we have highlighted the importance of single-cell observation methods for determining cancer cell responses to ionizing radiation and chemotherapeutic drugs, and have reviewed such assays, optimized by us [3,4,5], for assessment of cancer cell morphology, viability, and metabolic activity. In our view, these assays will be useful in identifying pharmacological agents capable of killing highly enlarged (e.g., polyploid/multinucleated) cancer cells before they will have the opportunity to enter the STAR cycle to promote tumor repopulation following anticancer treatment.

## References

[1] O’Neil N.J., Bailey M.L., Hieter P. (2017). Synthetic lethality and cancer. Nat. Rev. Genet..

[2] Mirzayans R., Andrais B., Murray D. (2018). Roles of polyploid/multinucleated giant cancer cells in metastasis and disease relapse following anticancer treatment. Cancers.

[3] Mirzayans R., Andrais B., Scott A., Wang Y.W., Kumar P., Murray D. (2017). Multinucleated giant cancer cells produced in response to ionizing radiation retain viability and replicate their genome. Int. J. Mol. Sci..

[4] Mirzayans R., Andrais B., Murray D. (2017). Impact of premature senescence on radiosensitivity measured by high throughput cell-based assays. Int. J. Mol. Sci..

[5] Mirzayans R., Andrais B., Murray D. (2017). Do multiwell plate high throughput assays measure loss of cell viability following exposure to genotoxic agents?. Int. J. Mol. Sci..

[6] Mirzayans R., Waters R. (1982). DNA damage and its repair in human normal or xeroderma pigmentosum fibroblasts treated with 4-nitroquinoline 1-oxide or its 3-methyl derivative. Carcinogenesis.

[7] Flusberg D.A., Sorger P.K. (2015). Surviving apoptosis: Life-death signaling in single cells. Trends Cell Biol..

[8] Mathiassen S.G., De Zio D., Cecconi F. (2017). Autophagy and the cell cycle: A complex landscape. Front. Oncol..

[9] Pérez-Garijo A. (2017). When dying is not the end: Apoptotic caspases as drivers of proliferation. Semin. Cell Dev. Biol..

[10] Niepel M., Spencer S.L., Sorger P.K. (2009). Non-genetic cell-to-cell variability and the consequences for pharmacology. Curr. Opin. Chem. Biol..

[11] Batchelor E., Loewer A. (2017). Recent progress and open challenges in modeling p53 dynamics in single cells. Curr. Opin. Syst. Biol..

[12] Albeck J.G., Burke J.M., Aldridge B.B., Zhang M., Lauffenburger D.A., Sorger P.K. (2008). Quantitative analysis of pathways controlling extrinsic apoptosis in single cells. Mol. Cell.

[13] Feinerman O., Veiga J., Dorfman J.R., Germain R.N., Altan-Bonnet G. (2008). Variability and robustness in T cell activation from regulated heterogeneity in protein levels. Science.

[14] Gascoigne K.E., Taylor S.S. (2008). Cancer cells display profound intra and interline variation following prolonged exposure to antimitotic drugs. Cancer Cell.

[15] Orth J.D., Tang Y., Shi J., Loy C.T., Amendt C., Wilm C., Zenke F.T., Mitchison T.J. (2008). Quantitative live imaging of cancer and normal cells treated with kinesin-5 inhibitors indicates significant differences in phenotypic responses and cell fate. Mol. Cancer Ther..

[16] Spencer S.L., Gaudet S., Albeck J.G., Burke J.M., Sorger P.K. (2009). Nongenetic origins of cell-to-cell variability in TRAIL-induced apoptosis. Nature.

[17] Irish J.M., Hovland R., Krutzik P.O., Perez O.D., Bruserud O., Gjertsen B.T., Nolan G.P. (2004). Single cell profiling of potentiated phospho-protein networks in cancer cells. Cell.

[18] Cohen A.A., Geva-Zatorsky N., Eden E., Frenkel-Morgenstern M., Issaeva I., Sigal A., Milo R., Cohen-Saidon C., Liron Y., Kam Z. (2008). Dynamic proteomics of individual cancer cells in response to a drug. Science.

[19] Geva-Zatorsky N., Rosenfeld N., Itzkovitz S., Milo R., Sigal A., Dekel E., Yarnitzky T., Liron Y., Polak P., Lahav G. (2006). Oscillations and variability in the p53 system. Mol. Syst. Biol..

[20] Kim C., Gao R., Sei E., Brandt R., Hartman J., Hatschek T., Crosetto N., Foukakis T., Navin N.E. (2018). Chemoresistance evolution in triple-negative breast cancer delineated by single-cell sequencing. Cell.

[21] Elmore S. (2007). Apoptosis: A review of programmed cell death. Toxicol. Pathol..

[22] Chen D.L., Engle J.T., Griffin E.A., Miller J.P., Chu W., Zhou D., Mach R.H. (2015). Imaging caspase-3 activation as a marker of apoptosis-targeted treatment response in cancer. Mol. Imaging Biol..

[23] Karamitopoulou E., Cioccari L., Jakob S., Vallan C., Schaffner T., Zimmermann A., Brunner T. (2007). Active caspase 3 and DNA fragmentation as markers for apoptotic cell death in primary and metastatic liver tumours. Pathology.

[24] Jonges L.E., Nagelkerke J.F., Ensink N.G., van der Velde E.A., Tollenaar R.A., Fleuren G.J., van de Velde C.J., Morreau H., Kuppen P.J. (2001). Caspase-3 activity as a prognostic factor in colorectal carcinoma. Lab. Investig..

[25] Zhou M., Liu X., Li Z., Huang Q., Li F., Li C.Y. (2018). Caspase-3 regulates the migration, invasion and metastasis of colon cancer cells. Int. J. Cancer.

[26] Pantel K., Brakenhoff R.H. (2004). Dissecting the metastatic cascade. Nat. Rev. Cancer.

[27] Polyak K., Weinberg R.A. (2009). Transitions between epithelial and mesenchymal states: Acquisition of malignant and stem cell traits. Nat. Rev. Cancer.

[28] Huang Q., Li F., Liu X., Li W., Shi W., Liu F.F., O’Sullivan B., He Z., Peng Y., Tan A.C. (2011). Caspase 3-mediated stimulation of tumor cell repopulation during cancer radiotherapy. Nat. Med..

[29] Liu X., He Y., Li F., Huang Q., Kato T.A., Hall R.P., Li C.Y. (2015). Caspase-3 promotes genetic instability and carcinogenesis. Mol. Cell.

[30] Kurtova A.V., Xiao J., Mo Q., Pazhanisamy S., Krasnow R., Lerner S.P., Chen F., Roh T.T., Lay E., Ho P.L. (2015). Blocking PGE2-induced tumour repopulation abrogates bladder cancer chemoresistance. Nature.

[31] Feng X., Yu Y., He S., Cheng J., Gong Y., Zhang Z., Yang X., Xu B., Liu X., Li C.Y. (2017). Dying glioma cells establish a proangiogenic microenvironment through a caspase 3 dependent mechanism. Cancer Lett..

[32] Flanagan L., Meyer M., Fay J., Curry S., Bacon O., Duessmann H., John K., Boland K.C., McNamara D.A., Kay E.W. (2016). Low levels of caspase-3 predict favourable response to 5FU-based chemotherapy in advanced colorectal cancer: Caspase-3 inhibition as a therapeutic approach. Cell Death Dis..

[33] Mirzayans R., Andrais B., Kumar P., Murray D. (2016). The growing complexity of cancer cell response to DNA-damaging agents: Caspase 3 mediates cell death or survival?. Int. J. Mol. Sci..

[34] Mirzayans R., Andrais B., Kumar P., Murray D. (2017). Significance of wild-type p53 signaling in suppressing apoptosis in response to chemical genotoxic agents: Impact on chemotherapy outcome. Int. J. Mol. Sci..

[35] Esmatabadi M.J.D., Bakhshinejad B., Motalegh F.M., Babashah S., Sadeghizadeh M. (2016). Therapeutic resistance and cancer recurrence mechanisms: Unfolding the story of tumour coming back. J. Biosci..

[36] Tang H.L., Yuen K.L., Tang H.M., Fung M.C. (2009). Reversibility of apoptosis in cancer cells. Br. J. Cancer.

[37] Tang H.L., Tang H.M., Mak K.H., Hu S., Wang S.S., Wong K.M., Wong C.S.T., Wu H.Y., Law H.T., Liu K. (2012). Cell survival, DNA damage, and oncogenic transformation after a transient and reversible apoptotic response. Mol. Biol. Cell.

[38] Tang H.L., Tang H.M., Fung M.C., Hardwick J.M. (2015). In vivo CaspaseTracker biosensor system for detecting anastasis and non-apoptotic caspase activity. Sci. Rep..

[39] Tang H.L., Tang H.M., Hardwick J.M., Fung M.C. (2015). Strategies for tracking anastasis, a cell survival phenomenon that reverses apoptosis. J. Vis. Exp. JoVE.

[40] Tang H.M., Talbot Jr. C.C., Fung M.C., Tang H.L. (2017). Molecular signature of anastasis for reversal of apoptosis. F1000Research.

[41] Sun G., Guzman E., Balasanyan V., Conner C.M., Wong K., Zhou H.R., Kosik K.S., Montell D.J. (2017). A molecular signature for anastasis, recovery from the brink of apoptotic cell death. J. Cell Biol..

[42] Riss T.L., Moravec R.A., Niles A.L., Duellman S., Benink H.A., Worzella T.J., Minor L., Sittampalam G.S., Coussens N.P., Brimacombe K. (2004). Cell Viability Assays.

[43] Riss T., O’Brien M., Moravec R. (2005). Selecting cell-based assays for drug discovery screening. Cell Notes.

[44] Berridge M.V., Herst P.M., Tan A.S. (2005). Tetrazolium dyes as tools in cell biology: New insights into their cellular reduction. Biotechnol. Annu. Rev..

[45] Liu Y., Peterson D.A., Kimura H., Schubert D. (1997). Mechanism of cellular 3-(4,5-dimethylthiazol-2-yl)-2,5-diphenyltetrazolium bromide (MTT) reduction. J. Neurochem..

[46] Berridge M.V., Tan A.S. (1993). Characterisation of the cellular reduction of 3-(4,5-dimethylthiazol-2yl)-2,5-diphenyltetrazolium bromide (MTT): Subcellular localization, substrate dependence, and involvement of mitochondrial electron transport in MTT reduction. Arch. Biochem. Biophys..

[47] Husmann M. (2013). Vital dyes and virtual deaths. Cell Death Differ..

[48] Rosenthal K., Oehling V., Dusny C., Schmid A. (2017). Beyond the bulk: Disclosing the life of single microbial cells. FEMS Microbiol. Rev..

[49] Rantalainen M. (2017). Application of single-cell sequencing in human cancer. Brief. Funct. Genom..

[50] Tellez-Gabriel M., Ory B., Lamoureux F., Heymann M.F., Heymann D. (2016). Tumour heterogeneity: The key advantages of single-cell analysis. Int. J. Mol. Sci..

[51] Chung W., Eum H.H., Lee H.O., Lee K.M., Lee H.B., Kim K.T., Ryu H.S., Kim S., Lee J.E., Park Y.H. (2017). Single-cell RNA-seq enables comprehensive tumour and immune cell profiling in primary breast cancer. Nat. Commun..

[52] Saadatpour A., Lai S., Guo G., Yuan G.H. (2015). Single-cell analysis in cancer genomics. Trends Genet..

[53] Eastman A. (2017). Improving anticancer drug development begins with cell culture: Misinformation perpetrated by the misuse of cytotoxicity assays. Oncotarget.

[54] Weihua Z., Lin Q., Ramoth A.J., Fan D., Fidler I.J. (2011). Formation of solid tumors by a single multinucleated cancer cell. Cancer.

[55] Erenpreisa J., Giuliani A., Vinogradov A.E., Anatskaya O.V., Vazquez-Martin A., Salmina K., Cragg M.S. (2018). Stress-induced polyploidy shifts somatic cells towards a pro-tumourogenic unicellular gene transcription network. Cancer Hypotheses.

[56] Thomas F., Ujvari B., Renaud F., Vincent M. (2017). Cancer adaptations: Atavism, de novo selection, or something in between?. Bioessays.

[57] Chen H., Lin F., Xing K., He X. (2015). The reverse evolution from multicellularity to unicellularity during carcinogenesis. Nat. Commun..

[58] Vincent M. (2016). Resistance to cancer chemotherapy as an atavism?. Bioessays.

[59] Niculescu V.F. (2018). Carcinogenesis: Recent insights in protist stem cell biology lead to a better understanding of atavistic mechanisms implied in cancer development. MOJ Tumor Res..

